# Supplementary material for: An Improved Phenotype-Driven Tool for Rare Mendelian Variant Prioritization: Benchmarking Exomiser on Real Patient Whole-Exome Data
Source: Genes (Basel). 2020 Apr 23;11(4):460. doi: 10.3390/genes11040460 (PMC7230372; doi:10.3390/genes11040460)
Supplement: Supplementary file 1 [file genes-11-00460-s001.pdf]

An Improved Phenotype-Driven Tool for Rare Mendelian Variant Prioritization: Benchmarking Exomiser on Real Patient Whole-Exome Data

Valentina Cipriani et al.

Supplementary Materials

Table S1. Molecular diagnosis (diagnosed gene, genotype and variant(s)) of the 134 patients in the IRD dataset.

		Molecular diagnosis			
**Patient identifier**	**Clinical diagnosis label[a]**	**Gene**	**Genotype[b]**	**Variant 1**	**Variant 2**
P1	ACHM	*CNGB3*	HOM	c.1810C>T:p.(Arg604*)	
P2	ACHM	*CNGB3*	HOM	c.1148del:p.(Thr383Ilefs*13)	
P3	ACHM	*ATF6*	HOM	c.970C>T:p.(Arg324Cys)	
P4	ACHM	*POC1B*	HOM	c.130_138del:p.(Ile44_Leu46del)	
P5	ACHM	*ATF6*	HOM	c.1187+5G>C:p.?	
P6	ACHM	*GNAT2*	HOM	c.906C>A:p.(Tyr302*)	
P7	BFR	*PLA2G5*	HOM	c.133G>T:p.(Gly45Cys)	
P8	COLOB	*YAP1*	HET	c.284T>C:p.(Phe95Ser)	
P9	CRD	*GUCA1A*	HET	c.332A>C:p.(Glu111Ala)	
P10	CRD	*AHI1*	compHET	c.2945G>T:p.(Arg982Met)	c.1558A>G:p.(Lys520Glu)
P11	CRD	*PROM1*	HET	c.1117C>T:p.(Arg373Cys)	

P12	CRD	*PROM1*	HOM	c.436C>T:p.(Arg146*)	
P13	CRD	*RPGR*	HEMI	c.3039_3040del:p.(Glu1018Argfs*60)	
P14	CRD	*ABCA4*	compHET	c.2861A>C:p.(Tyr954Ser)	c.3191-1G>T:p.?
P15	CRD	*MERTK*	HOM	c.1470del:p.(Pro490Profs*25)	
P16	CRD	*TTLL5*	HOM	c.1627G>T:p.(Glu543*)	
P17	CRD	*CERKL*	HOM	c.316C>A:p.(Arg106Ser)	
P18	CRD	*CFAP410*	HOM	c.218G>C:p.(Arg73Pro)	
P19	CRD	*AGBL5*	HOM	c.323C>G:p.(Pro108Arg)	
P20	CRD	*CFAP410*	compHET	c.218G>C:p.(Arg73Pro)	c.655A>T:p.(Ile219Phe)
P21	CRD	*CDHR1*	HOM	c.1463del:p.(Gly488Alafs*20)	
P22	CRD	*CDHR1*	HOM	c.1463del:p.(Gly488Alafs*20)	
P23	CSNB	*NYX*	HEMI	c.998_1003del:p.(Leu333_Phe334del)	
P24	CSNB	*TRPM1*	compHET	c.380G>A:p.(Gly127Glu)	c.832G>A:p.(Gly278Arg)
P25	CSNB	*GPR179*	compHET	c.870dup:p.(Asn291*)	c.1368del:p.(Phe456Leufs*30)
P26	CSNB	*RBP3*	HOM	c.3454G>T:p.(Glu1152*)	
P27	CSNB	*TRNT1*	HOM	c.295C>T:p.(Arg99Trp)	
P28	EORD	*LCA5*	HOM	c.617T>C:p.(Leu206Pro)	
P29	EORD	*PDE6A*	HOM	c.769C>T:p.(Arg257*)	
P30	EORD	*BBS1*	HOM	c.200G>A:p.(Arg67Glnext*-67)	
P31	EORD	*CDH3*	HOM	c.2357del:p.(Gly786Alafs*7)	
P32	EORD	*CRX*	HET	c.624T>A:p.(Tyr208*)	
P33	EORD	*IMPG2*	HOM	c.1875_1879dup:p.(Pro627Leufs*25)	
P34	EORD	*ADAM9*	HOM	c.967del:p.(Ser323Glnfs*33)	
P35	EORD	*IQCB1*	compHET	c.825_828del:p.(Arg275Serfs*6)	c.745A>T:p.(Arg249*)
P36	EORD	*CEP290*	HOM	c.2450T>G:p.(Ile817Ser)	
P37	FEVR	*LRP5*	compHET	c.1435G>A:p.(Gly479Arg)	c.4097A>G:p.(Asp1366Gly)
P38	FH	*HPS6*	HOM	c.779G>A:p.(Gly260Glu)	
P39	LCA	*ABCA4*	compHET	c.4918C>T:p.(Arg1640Trp)	c.2041C>T:p.(Arg681*)
P40	LCA	*GUCY2D*	HOM	c.2120T>C:p.(Leu707Pro)	
P41	LCA	*COL18A1*	HOM	c.714dup:p.(Gly239Argfs*9)	

P42	LCA	*GUCY2D*	HOM	c.652del:p.(Met218Trpfs*13)	
P43	LCA	*DHX38*	HOM	c.971G>A:p.(Arg324Gln)	
P44	LCA	*SRD5A3*	HOM	c.57G>A:p.(Trp19*)	
P45	LCA	*GUCY2D*	HOM	c.2836G>A:p.(Ala946Thr)	
P46	LCA	*RPGRIP1*	compHET	c.711del:p.(Pro237Profs*38)	c.2786A>G:p.(Tyr929Cys)
P47	LCA	*CEP290*	HOM	c.148C>T:p.(His50Tyr)	
P48	LCA	*CRB1*	compHET	c.976T>C:p.(Cys326Arg)	c.1798C>T:p.(Gln600*)
P49	LCA	*NMNAT1*	HOM	c.53A>G:p.(Asn18Ser)	
P50	LCA	*CLN3*	HOM	c.932C>A:p.(Ser311Tyr)	
P51	LCA	*TUB*	HOM	c.1194_1195del:p.(Arg398Serfs*10)	
P52	LCA	*KCNJ13*	HOM	c.496C>T:p.(Arg166*)	
P53	LCA	*LCA5*	HOM	c.1676C>A:p.(Ser559*)	
P54	LCA	*RP1*	HOM	c.1458_1461dup:p.(Glu488*)	
P55	LCA	*BBS1*	HOM	c.200G>A:p.(Arg67Glnext*-67)	
P56	LCA	*IMPG2*	HOM	c.68dup:p.(Asp23Glufs*29)	
P57	LCA	*IFT140*	HOM	c.1451C>T:p.(Thr484Met)	
P58	LCA	*SPATA7*	HOM	c.864dup:p.(Pro289Thrfs*6)	
P59	LCA	*GUCY2D*	HOM	c.2395_2398dup:p.(His800Argfs*20)	
P60	LCA	*TULP1*	HOM	c.751G>T:p.(Glu251*)	
P61	LCA	*IQCB1*	HOM	c.1363C>T:p.(Arg455*)	
P62	LCA	*ABCA4*	compHET	c.161G>A:p.(Cys54Tyr)	c.2160+1G>C:p.?
P63	LCA	*GUCY2D*	HOM	c.2828dup:p.(Arg944Alafs*27)	
P64	MD	*MFSD8*	HOM	c.1361T>C:p.(Met454Thr)	
P65	MD	*CRX*	HET	c.774T>A:p.(Tyr258*)	
P66	MD	*GUCY2D*	HET	c.2512C>T:p.(Arg838Cys)	
P67	MD	*GUCY2D*	HET	c.2512C>T:p.(Arg838Cys)	
P68	MD	*CRX*	HET	c.121C>T:p.(Arg41Trp)	
P69	MD	*RPGR*	HEMI	c.3317dup:p.(Ser1107Valfs*4)	
P70	MD	*CRX*	HET	c.127C>T:p.(Arg43Cys)	
P71	MD	*ABCA4*	compHET	c.3522G>A:p.(=)	c.5527C>G:p.(Arg1843Gly)

P72	MD	*CRX*	HET	c.272G>A:p.(Arg91Lys)	
P73	MD	*ABCA4*	compHET	c.5882G>A:p.(Gly1961Glu)	c.885del:p.(Asp295Aspfs*5)
P74	MD	*DRAM2*	HOM	c.362A>T:p.(His121Leu)	
P75	MD	*TTLL5*	HOM	c.1586_1589del:p.(Glu529Valfs*2)	
P76	MD	*RPGR*	HEMI	c.3178_3179del:p.(Glu1066Glyfs*12)	
P77	MD	*CDH3*	HOM	c.1568del:p.(Asn523Metfs*14)	
P78	MD	*TTLL5*	compHET	c.401del:p.(Leu134Argfs*45)	c.3354G>A:p.(Trp1118*)
P79	MD	*DRAM2*	compHET	c.217_225del:p.(Val73_Tyr75del)	c.79T>C:p.(Tyr27His)
P80	STICKL	*COL9A3*	HOM	c.1739dup:p.(Gly581Trpfs*20)	
P81	OCMD	*MFSD8*	compHET	c.103C>T:p.(Arg35*)	c.1006G>C:p.(Glu336Gln)
P82	OALB	*GPR143*	HEMI	c.839A>G:p.(Asn280Ser)	
P83	OATR	*KIF11*	HET	c.247C>T:p.(Arg83*)	
P84	RD	*EYS*	compHET	c.7994G>A:p.(Gly2665Glu)	c.2976T>A:p.(Cys992*)
P85	RD	*LCA5*	HOM	c.633_639del:p.(Glu211Aspfs*13)	
P86	RD	*CERKL*	HOM	c.316C>A:p.(Arg106Ser)	
P87	RP	*PDE6A*	HOM	c.1630C>T:p.(Arg544Trp)	
P88	RP	*RPGR*	HEMI	c.126T>G:p.(Cys42Trp)	
P89	RP	*USH2A*	compHET	c.2299del:p.(Glu767Serfs*21)	c.6050-1G>A:p.?
P90	RP	*USH2A*	compHET	c.7334C>T:p.(Ser2445Phe)	c.3902G>T:p.(Gly1301Val)
P91	RP	*IMPG2*	compHET	c.2426G>A:p.(Trp809*)	c.3412_3413insAA:p.(Ser1138Lysfs*21)
P92	RP	*CNGB1*	compHET	c.952C>T:p.(Gln318*)	c.3A>T:p.0?
P93	RP	*PDE6A*	compHET	c.1630C>T:p.(Arg544Trp)	c.769C>T:p.(Arg257*)
P94	RP	*RPGR*	HEMI	c.2323_2324del:p.(Arg775Glufs*59)	
P95	RP	*CERKL*	HOM	c.847C>T:p.(Arg283*)	
P96	RP	*IMPG2*	compHET	c.2426G>A:p.(Trp809*)	c.118G>T:p.(Glu40*)
P97	RP	*RPGR*	HEMI	c.2236_2237del:p.(Lys751Glyfs*18)	
P98	RP	*RP1*	HET	c.2206dup:p.(Thr736Asnfs*4)	
P99	RP	*BBS1*	HOM	c.1169T>G:p.(Met390Arg)	
P100	RP	*RPGR*	HEMI	c.914dup:p.(Asn305Lysfs*41)	
P101	RP	*CRB1*	HOM	c.1337T>C:p.(Ile446Thr)	

P102	RP	*RP1*	HOM	c.5883del:p.(Gln1961Glnfs*16)	
P103	RP	*CNGB1*	compHET	c.664C>T:p.(Gln222*)	c.262C>T:p.(Gln88*)
P104	RP	*BBS1*	HOM	c.1169T>G:p.(Met390Arg)	
P105	RP	*USH2A*	compHET	c.9882C>G:p.(Cys3294Trp)	c.653T>A:p.(Val218Glu)
P106	RP	*USH2A*	compHET	c.13274C>T:p.(Thr4425Met)	c.8981G>A:p.(Trp2994*)
P107	RP	*CRB1*	HOM	c.782A>G:p.(Asn261Ser)	
P108	RP	*RPGR*	HEMI	c.2250_2251del:p.(Lys751Glyfs*18)	
P109	RP	*AHI1*	compHET	c.2429C>T:p.(Pro810Leu)	c.2087A>G:p.(His696Arg)
P110	RP	*EYS*	HOM	c.490C>T:p.(Arg164*)	
P111	RP	*CRB1*	HOM	c.1819G>T:p.(Gly607*)	
P112	RP	*CNGB1*	compHET	c.3139_3142dup:p.(Ala1048Glyfs*13)	c.3A>T:p.0?
P113	RP	*CNGB1*	HOM	c.3A>T:p.0?	
P114	RP	*BBS1*	HOM	c.200G>A:p.(Arg67Glnext*-67)	
P115	RP	*PDE6B*	HOM	c.1485dup:p.(Pro496Alafs*5)	
P116	RP	*CNGB1*	HOM	c.761+2T>A:p.?	
P117	RP	*AHI1*	compHET	c.2090C>T:p.(Pro697Leu)	c.660del:p.(Pro220Profs*11)
P118	RP	*RP1L1*	HOM	c.603del:p.(Gly201Glyfs*30)	
P119	RP	*ARL2BP*	HOM	c.134T>G:p.(Met45Arg)	
P120	RP	*ABCA4*	HOM	c.3393del:p.(Ala1131Alafs*17)	
P121	RP	*PROM1*	HOM	c.2346del:p.(Phe782Phefs*10)	
P122	RP	*TULP1*	compHET	c.1081C>T:p.(Arg361*)	c.1255C>T:p.(Arg419Trp)
P123	STGD	*ABCA4*	HOM	c.6658C>T:p.(Gln2220*)	
P124	STGD	*ABCA4*	compHET	c.3364G>A:p.(Glu1122Lys)	c.1906C>T:p.(Gln636*)
P125	USH1	*MYO7A*	compHET	c.598A>G:p.(Ser200Gly)	c.611A>C:p.(Lys204Thr)
P126	USH1	*ADGRV1*	compHET	c.13919G>A:p.(Gly4640Glu)	c.18610G>A:p.(Gly6204Ser)
P127	USH2	*USH2A*	compHET	c.3832_3834del:p.(Leu1278del)	c.920_923dup:p.(His308Glnfs*16)
P128	USH2	*USH2A*	HOM	c.2299del:p.(Glu767Serfs*21)	
P129	USH2	*USH2A*	compHET	c.1055C>T:p.(Thr352Ile)	c.12819T>A:p.(Tyr4273*)
P130	USH2	*ADGRV1*	HET	c.8807C>G:p.(Ser2936*)	
P131	USH2	*USH2A*	compHET	c.12954C>A:p.(Tyr4318*)	c.5603T>G:p.(Phe1868Cys)

P132	USH2	*USH2A*	compHET	c.7594+2T>A:p.?	c.4632G>C:p.(Lys1544Asn)
P133	USH2	*ADGRV1*	compHET	c.3290-1G>A:p.?	c.11242dup:p.(Glu3748Glyfs*10)
P134	USH2	*USH2A*	compHET	c.5012G>A:p.(Gly1671Asp)	c.5252G>T:p.(Gly1751Val)

[a]Clinical diagnosis as assigned by a consultant ophthalmologist before performing whole-exome sequencing.

[b]HET = heterozygote; HOM = homozygote; compHET = compound heterozygote; HEMI = hemizygote.

Table S2. List of HPO terms used to encode the 19 clinical diagnoses observed in the IRD patient dataset.

Clinical diagnosis label	HPO terms	HPO term description
ACHM	HP:0011516, HP:0008275	Achromatopsia; Abnormal light-adapted electroretinogram
BFR	HP:0012045	Retinal flecks
COLOB	HP:0000480	Retinal coloboma
CRD	HP:0000548	Cone/Cone-rod dystrophy
CSNB	HP:0007642, HP:0007984	Congenital stationary night blindness; Electronegative ERG
EORD	HP:0000556, HP:0011463	Retinal dystrophy; Childhood onset
FEVR	HP:0030490	Exudative vitreoretinopathy
FH	HP:0007750	Hypoplasia of the fovea
LCA	HP:0000556, HP:0007758, HP:0000550, HP:0000639	Retinal dystrophy; Visual impairment; Undetectable electroretinogram; Nystagmus
MD	HP:0007754	Macular dystrophy
STICKL	HP:0000545, HP:0008527	Myopia; Congenital sensorineural hearing impairment
OCMD	HP:0030636, HP:0030488	Occult macular dystrophy; Abnormal central response of multifocal electroretinogram
OALB	HP:0001107, HP:0007730, HP:0007750, HP:0030464	Ocular albinism; Iris hypopigmentation; Hypoplasia of the fovea; Asymmetrical distribution of pattern reversal visual evoked potentials
OATR	HP:0001138, HP:0000649, HP:0030487	Optic neuropathy; Abnormality of visual evoked potentials; Abnormal P50/N95 ratio of pattern electroretinogram
RD	HP:0000556	Retinal dystrophy
RP	HP:0000510	Rod-cone dystrophy
STGD	HP:0007754	Macular dystrophy
USH1	HP:0000510, HP:0008527, HP:0011476, HP:0007642, HP:0001751, HP:0000654	Rod-cone dystrophy; Congenital sensorineural hearing impairment; Profound sensorineural hearing impairment; Congenital stationary night blindness; Vestibular dysfunction; Decreased light- and dark-adapted electroretinogram amplitude
USH2	HP:0000510, HP:0008527, HP:0012712, HP:0007642, HP:0000654	Rod-cone dystrophy; Congenital sensorineural hearing impairment; Mild hearing impairment; Congenital stationary night blindness; Decreased light- and dark-adapted electroretinogram amplitude

Table S3. Exomiser ranking of the correct disease-causing variants for the 4 cases in the IRD patient dataset who received a new (syndromic) diagnosis following whole-exome sequencing.

						Exomiser rank using different analysis settings							
**Patient identifier**	**Initial diagnosis (before WES)**	**New diagnosis (after WES)**	**Gene**	**Diagnosed variant**	**Genotype**	**DEFAULT**	**CADD**	**REVEL**	**MPC**	**M_CAP**	**MVP**	**PRIMATE_AI**	**VAR-ONLY**
P38	Foveal hypoplasia	Hermansky-Pudlak syndrome	*HPS6*	c.779G>A:p.(Gly260Glu)	HOM	1	1	1	17	25	1	1	7
P41	Leber congenital amaurosis	Knobloch syndrome	*COL18A1*	c.714dup:p.(Gly239Argfs*9)	HOM	2	2	1	1	1	1	1	4
P44	Leber congenital amaurosis	*SRD5A3*-related congenital disorder of glycosylation	*SRD5A3*	c.57G>A:p.(Trp19*)	HOM	1	1	1	1	1	1	1	3.5
P50	Leber congenital amaurosis	Batten disease	*CLN3*	c.932C>A:p.(Ser311Tyr)	HOM	1	1	1	1	1	1	1	3

WES = Whole-Exome Sequencing

Table S4. Exomiser ranking of the correct disease-causing variants for the 5 IRD patients with rank > 5 using the DEFAULT analysis settings.

Patient identifier	Gene	Diagnosed variant	Genotype	Exomiser rank using DEFAULT analysis settings	Exomiser variant score	Exomiser phenotype score	Exomiser phenotype matches
P43	*DHX38*	c.971G>A:p.(Arg324Gln)	HOM	7	0.997	0.502	**Proximity score 0.502 in interactome to *AAAS* and phenotypic similarity 0.617 to Achalasia-addisonianism-alacrimia syndrome associated with *AAAS***<br>Best Phenotype Matches:<br>**HP:0000556**, Retinal dystrophy - HP:0000648, Optic atrophy<br>**HP:0000505**, Visual impairment – *no matches*<br>**HP:0000550**, Undetectable electroretinogram - HP:0000649, Abnormality of visual evoked potentials<br>**HP:0000639**, Nystagmus – *no matches*<br><br>**No known disease**
P4	*POC1B*	c.130_138del:p.(Ile44_Leu46del)	HOM	9	0.848	0.587	**Phenotypic similarity 0.587 to Cone rod dystrophy associated with *POC1B***<br>Best Phenotype Matches:<br>**HP:0011516**, Achromatopsia - HP:0000551, Abnormality of color vision<br>**HP:0008275**, Abnormal light-adapted electroretinogram – *no matches*<br><br>**Known diseases:**<br>OMIM:615973 Cone-rod dystrophy 20 - autosomal recessive<br>ORPHA:1872 Cone rod dystrophy

P27	*TRNT1*	c.295C>T:p.(Arg99Trp)	HOM	12	0.989	0.500	**Phenotypic similarity 0.476 to Retinitis pigmentosa and erythrocytic microcytosis associated with *TRNT1***<br>Best Phenotype Matches:<br>**HP:0007642**, Congenital stationary night blindness - HP:0000662, Nyctalopia<br>**HP:0007984**, Electronegative electroretinogram – *no matches*<br><br>**Proximity score 0.500 in interactome to *RP9* and phenotypic similarity 0.622 to ?Retinitis pigmentosa 9 associated with *RP9***<br>Best Phenotype Matches:<br>**HP:0007642**, Congenital stationary night blindness - HP:0000662, Nyctalopia<br>**HP:0007984**, Electronegative electroretinogram - HP:0007688, Undetectable light- and dark-adapted electroretinogram<br><br>**Known diseases:**<br>OMIM:616084 Sideroblastic anemia with B-cell immunodeficiency, periodic fevers, and developmental delay - autosomal recessive<br>OMIM:616959 Retinitis pigmentosa and erythrocytic microcytosis - autosomal recessive
P80	*COL9A3*	c.1739dup:p.(Gly581Trpfs*20)	HOM	39	0.997	0.378	**Phenotypic similarity 0.755 to Autosomal recessive Stickler syndrome associated with *COL9A3***<br>Best Phenotype Matches:<br>**HP:0000545**, Myopia - HP:0000545, Myopia<br>**HP:0008527**, Congenital sensorineural hearing impairment - HP:0000407, Sensorineural hearing impairment<br><br>**Proximity score 0.506 in interactome to *COL9A2* and phenotypic similarity 0.755 to Autosomal recessive Stickler syndrome associated with *COL9A2***<br>Best Phenotype Matches:

							**HP:0000545**, Myopia - HP:0000545, Myopia<br>**HP:0008527**, Congenital sensorineural hearing impairment - HP:0000407, Sensorineural hearing impairment<br><br>**Proximity score 0.506 in interactome to *COL9A2* and phenotypic similarity 0.388 to mouse mutant of *COL9A2***<br>Best Phenotype Matches:<br>**HP:0000545**, Myopia – *no matches*<br>**HP:0008527**, Congenital sensorineural hearing impairment - MP:0011967, increased or absent threshold for auditory brainstem response<br><br>**Known diseases - observed variants incompatible with mode of inheritance:**<br>OMIM:600969 Epiphyseal dysplasia, multiple, 3, with or without myopathy - autosomal dominant<br>ORPHA:166002 Multiple epiphyseal dysplasia due to collagen 9 anomaly<br>ORPHA:250984 Autosomal recessive Stickler syndrome
P118	*RP1L1*	c.603del:p.(Gly201Glyfs*30)	HOM	42	0.996	0.405	**Phenotypic similarity 0.809 to Occult macular dystrophy associated with *RP1L1***<br>Best Phenotype Matches:<br>**HP:0000510**, Rod-cone dystrophy - HP:0007754, Macular dystrophy<br><br>**Phenotypic similarity 0.695 to mouse mutant involving *RP1L1***<br>Best Phenotype Matches:<br>**HP:0000510**, Rod-cone dystrophy - MP:0001325, abnormal retina morphology<br><br>**Known diseases - observed variants incompatible with mode of inheritance:**<br>OMIM:613587 Occult macular dystrophy - autosomal dominant

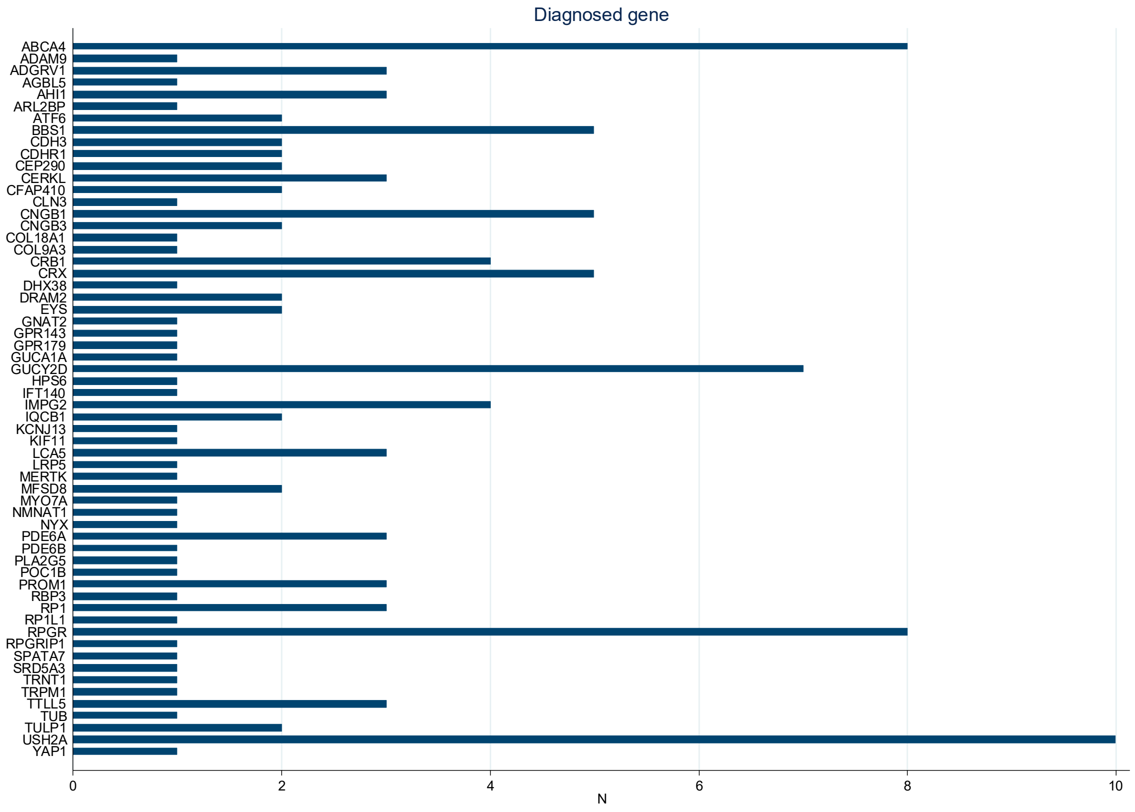

Figure S1. Frequency distribution of the 60 diagnosed genes in the IRD patient dataset (N=134).

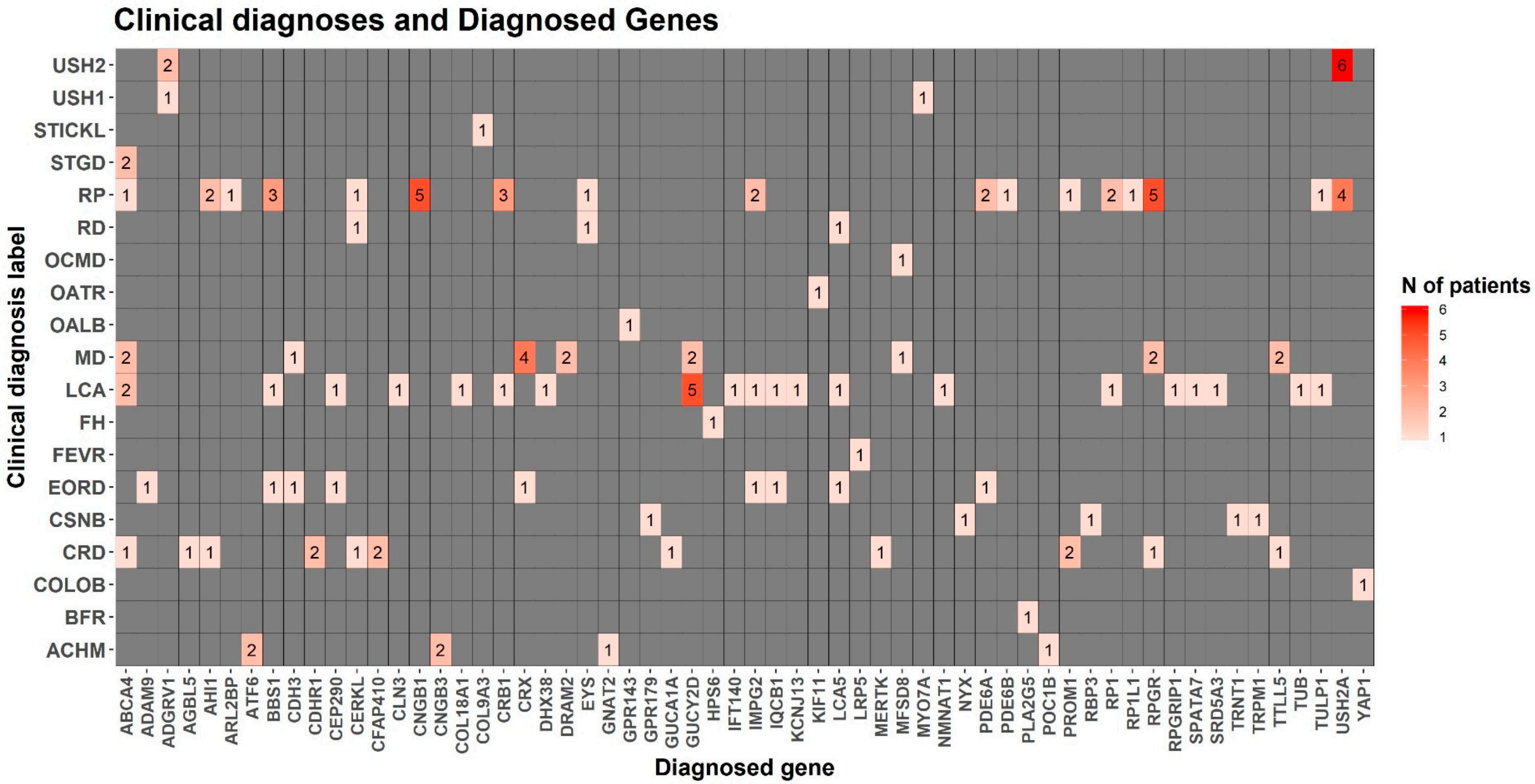

Figure S2. Clinical diagnoses and diagnosed genes in the IRD patient dataset (N=134). The figure shows the number of patients per each clinical diagnosis (y-axis) and each diagnosed gene (x-axis). For example, mutations in *CRB1* caused LCA (Leber congenital amaurosis) and RP (Retinitis pigmentosa) (phenotypic heterogeneity) in a total of 4 patients. On the other hand, ACHM (Achromatopsia) was found to be caused by mutations in 4 different genes, i.e., *ATF6*, *CNGB3*, *GNAT2* and *POC1B* (genetic heterogeneity) in a total of 6 patients.

Retinitis pigmentosa (RP) (*Rod-cone dystrophy*, HP:0000510)

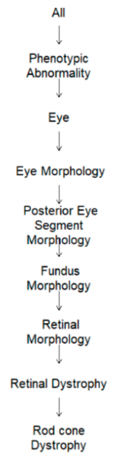

Usher syndrome type II (USH2) (*Rod-cone dystrophy,* HP:0000510; *Congenital sensorineural hearing impairment*, HP:0008527; *Mild hearing impairment*, HP:0012712; *Congenital stationary night blindness*, HP:0007642; *Decreased light- and dark-adapted electroretinogram amplitude*, HP:0000654)

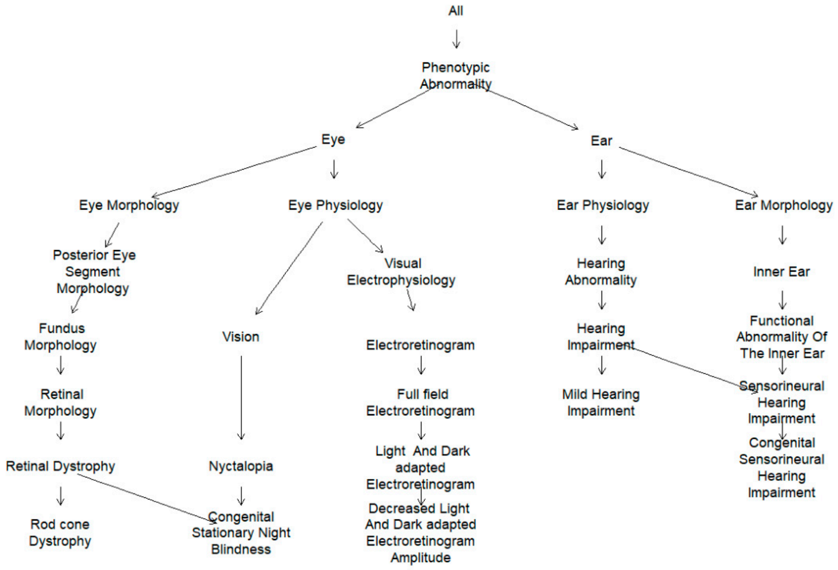

Figure S3. HPO graphic visualization of the 19 clinical diagnoses in the IRD patient dataset.

(continued on the next page)

Leber congenital amaurosis (LCA) (*Retinal dystrophy*, HP:0000556; *Visual impairment*, HP:0007758; *Undetectable electroretinogram*, HP:0000550; *Nystagmus*, HP:0000639)

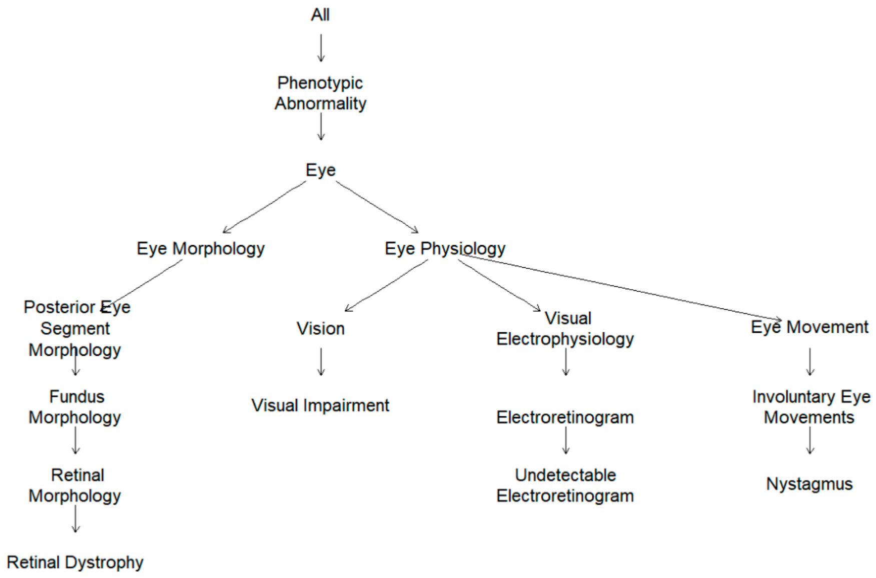

Stargardt disease (STGD) (*Macular dystrophy*, HP:0007754)

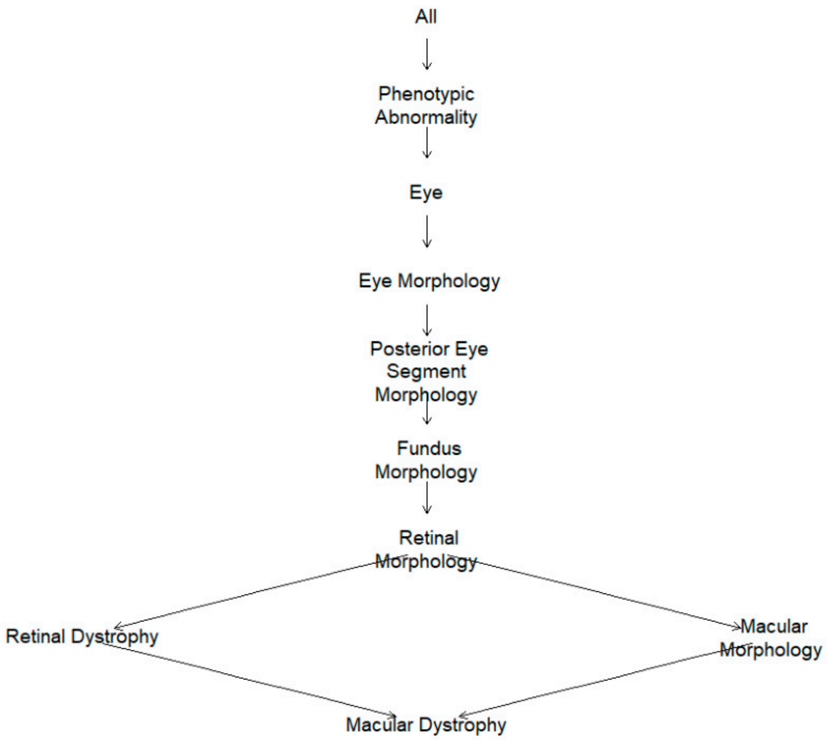

(continued on the next page)

Congenital stationary night blindness (CSNB) (*Congenital stationary night blindness*, HP:0007642; *Electronegative ERG*, HP:0007984)

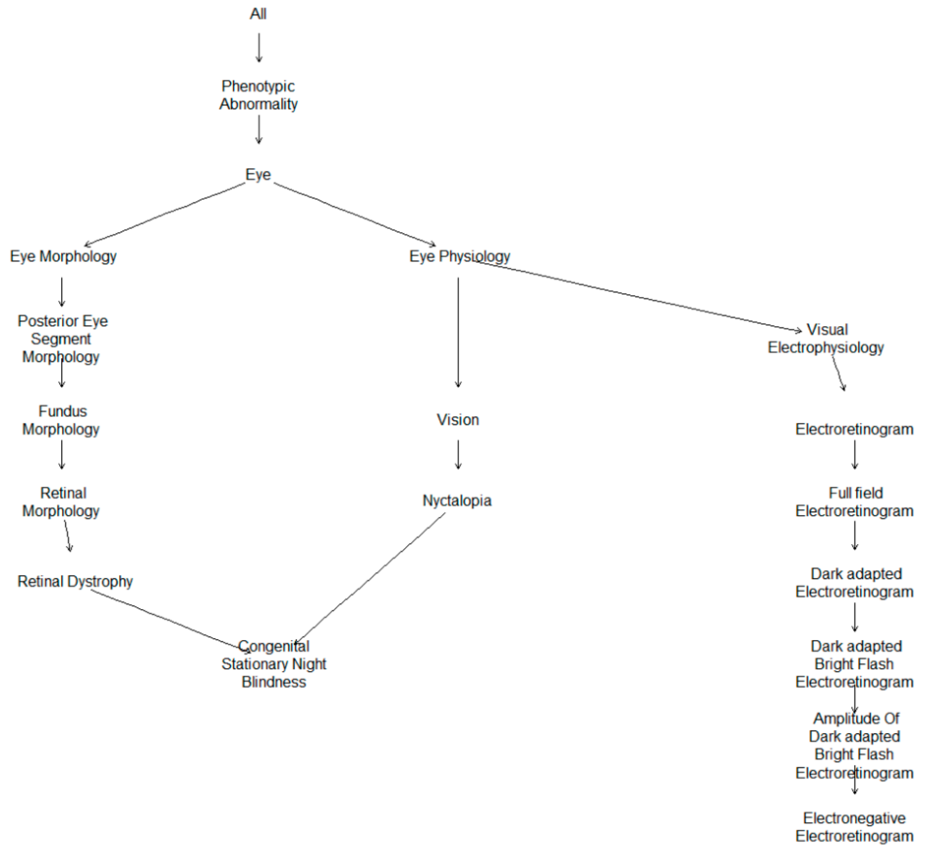

Retinal dystrophy (RD) (*Retinal dystrophy*, HP:0000556)

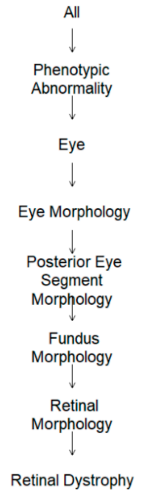

(continued on the next page)

Cone-rod dystrophy (CRD) (*Cone/Cone-rod dystrophy*, HP:0000548)

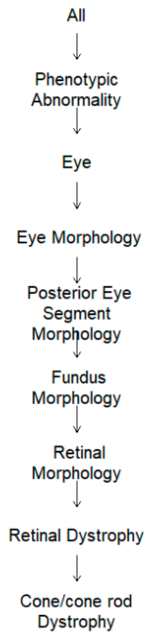

Achromatopsia (ACHM) (*Achromatopsia*, HP:0011516; *Abnormal light-adapted electroretinogram*, HP:0008275)

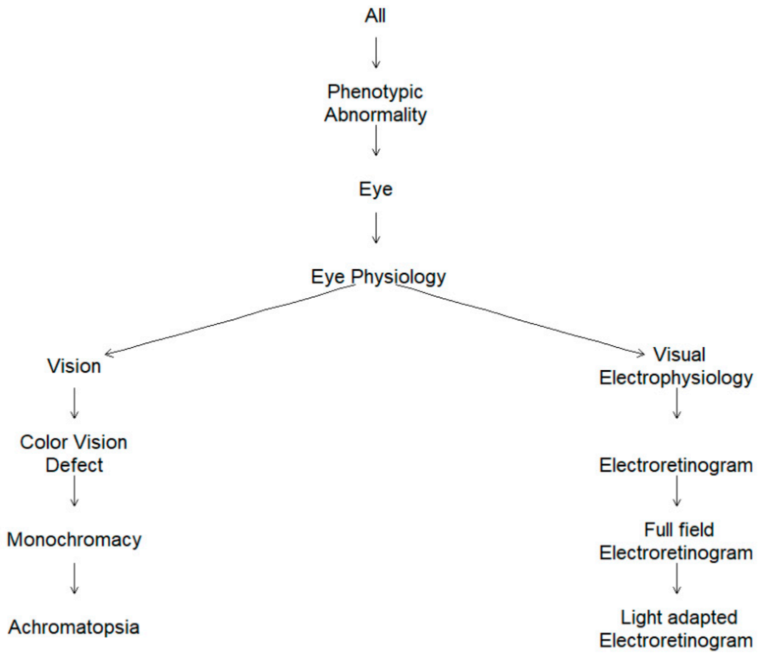

(continued on the next page)

Ocular albinism (OALB) (*Ocular albinism*, HP:0001107; *Iris hypopigmentation*, HP:0007730; *Hypoplasia of the fovea*, HP:0007750; *Asymmetrical distribution of pattern reversal visual evoked potentials*, HP:0030464)

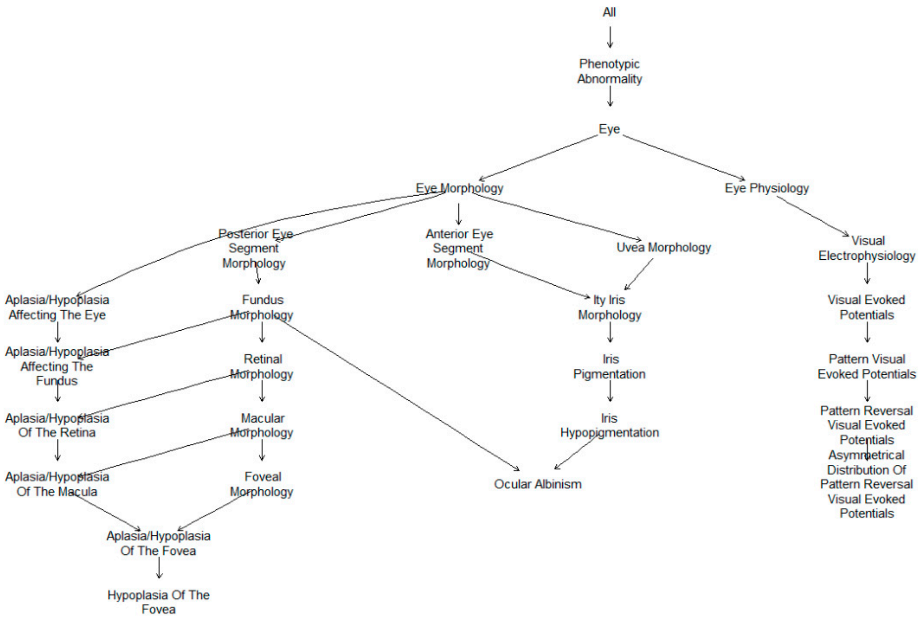

Macular dystrophy (MD) (*Macular dystrophy*, HP:0007754)

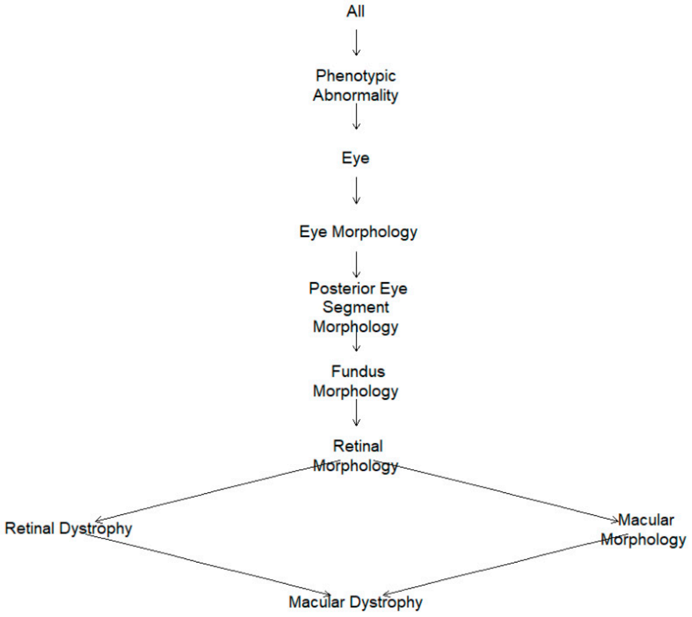

(continued on the next page)

Early onset retinal dystrophy (EORD) (*Retinal dystrophy*, HP:0000556; *Childhood onset*, HP:0011463)

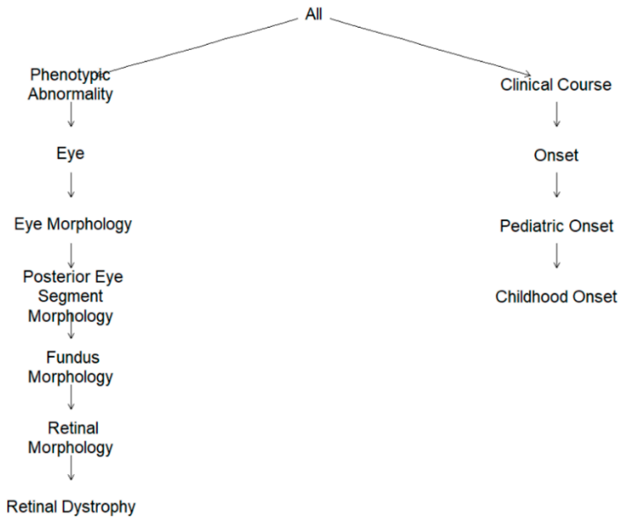

Foveal hypoplasia (FH) (*Hypoplasia of the fovea*, HP:0007750)

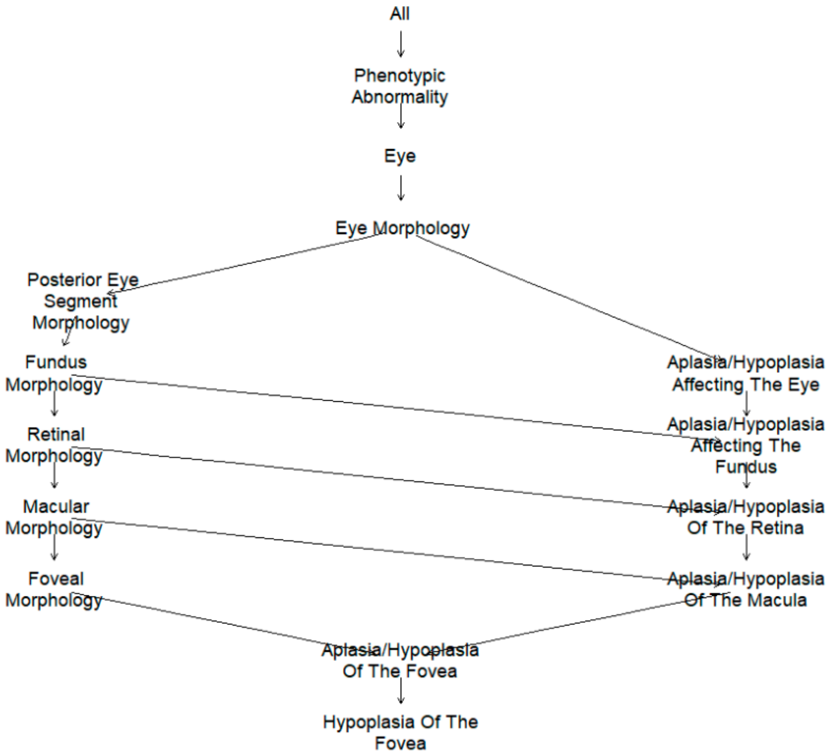

(continued on the next page)

Optic atrophy (OATR) (*Optic neuropathy*, HP:0001138; *Abnormality of visual evoked potentials*, HP:0000649; *Abnormal P50/N95 ratio of pattern electroretinogram*, HP:0030487)

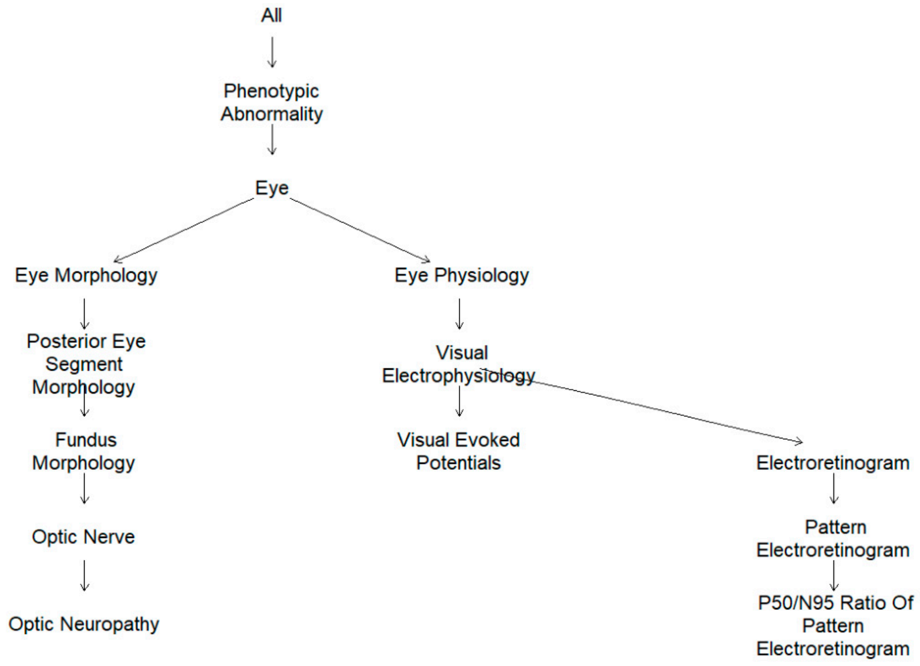

Familial exudative vitreoretinopathy (FEVR) (*Exudative vitreoretinopathy*, HP:0030490)

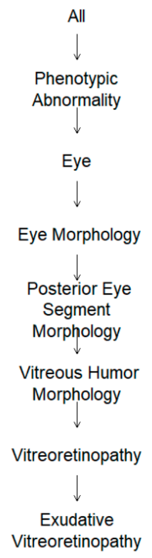

Benign fleck retina (BFR) (*Retinal flecks*, HP:0012045)

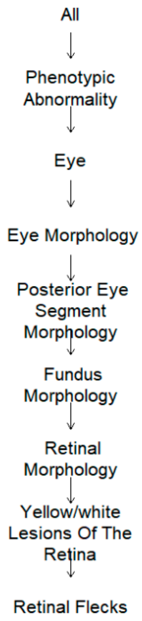

(continued on the next page)

Occult macular dystrophy (OCMD) (*Occult macular dystrophy*, HP:0030636; *Abnormal central response of multifocal electroretinogram*, HP:0030488)

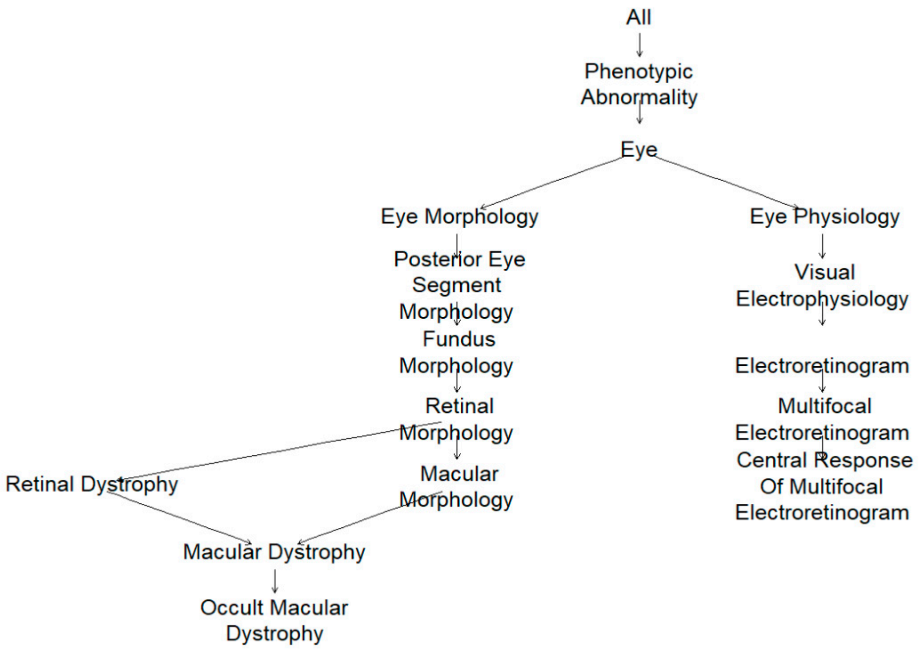

Coloboma (COLOB) (*Retinal coloboma*, HP:0000480)

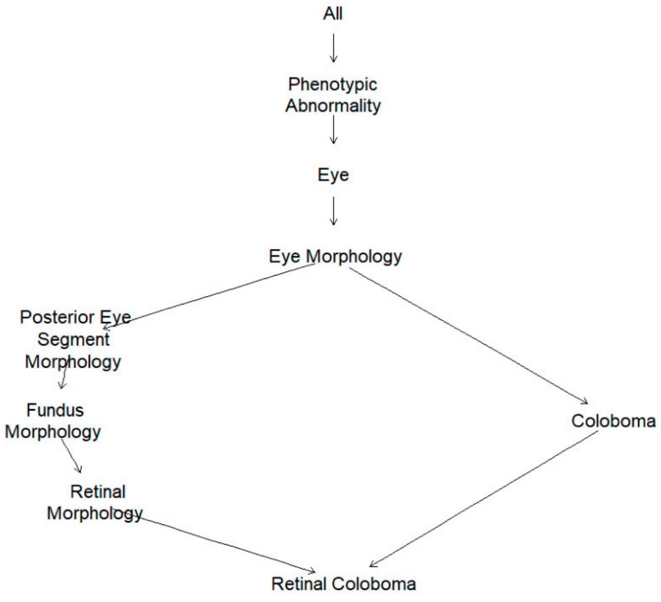

(continued on the next page)

Myopia and deafness (Stickler syndrome) (STICKL) (*Myopia*, HP:0000545; *Congenital sensorineural hearing impairment*, HP:0008527)

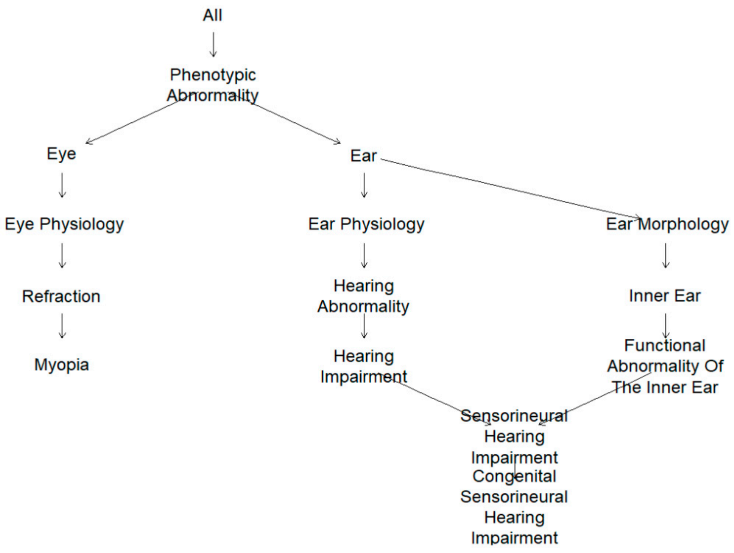

Usher syndrome type I (USH1) (*Rod-cone dystrophy*, HP:0000510; *Congenital sensorineural hearing impairment*, HP:0008527; *Profound sensorineural hearing impairment*, HP:0011476; *Congenital stationary night blindness*, HP:0007642; *Vestibular dysfunction*, HP:0001751; *Decreased light- and dark-adapted electroretinogram amplitude*, HP:0000654)

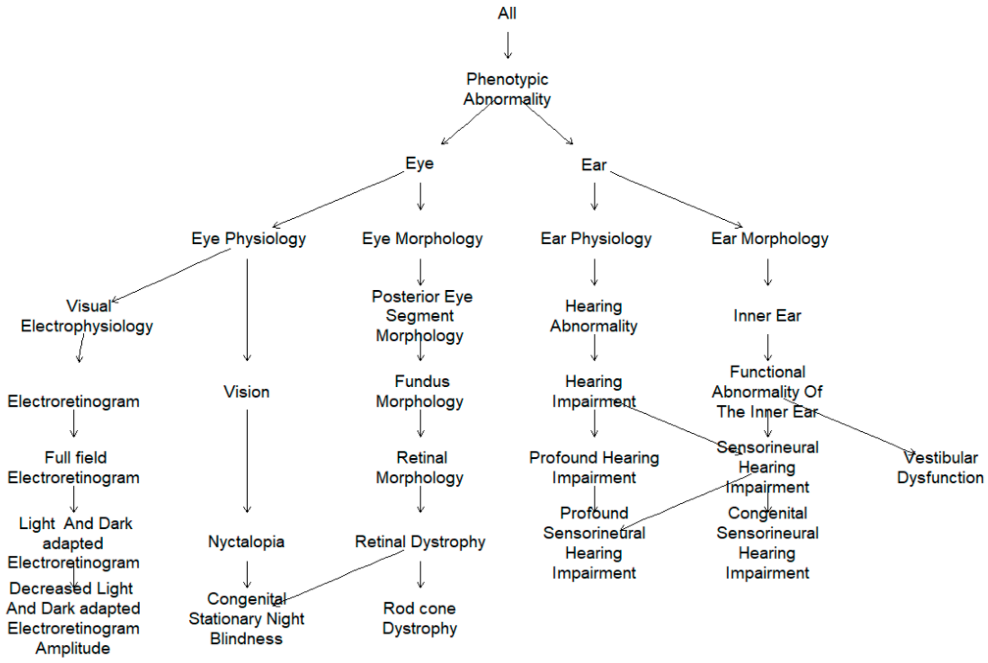

ABCA4 Exomiser Score: **0.998** Phenotype Score: **1.000** Variant Score: **0.999**

Phenotype matches:
Phenotypic similarity 1.000 to Cone-rod dystrophy 3 associated with ABCA4.
Best Phenotype Matches:
HP:0000548, Cone/cone-rod dystrophy - HP:0000548, Cone/cone-rod dystrophy

Phenotypic similarity 0.571 to mouse mutant involving ABCA4.
Best Phenotype Matches:
HP:0000548, Cone/cone-rod dystrophy - MP:0005201, abnormal retinal pigment epithelium morphology

Proximity score 0.570 in interactome to IMPG1 and phenotypic similarity 0.693 to Macular dystrophy, vitelliform, 4 associated with IMPG1.
Best Phenotype Matches:
HP:0000548, Cone/cone-rod dystrophy - HP:0007754, Macular dystrophy

Known diseases:
OMIM:153800 Macular degeneration, age-related, 2 (susceptibility)
OMIM:248200 Fundus flavimaculatus - autosomal recessive
OMIM:601718 Retinitis pigmentosa 19 - autosomal recessive
OMIM:604116 Cone-rod dystrophy 3 - unknown
ORPHA:1872 Cone rod dystrophy
ORPHA:791 Retinitis pigmentosa
ORPHA:827 Stargardt disease

AUTOSOMAL_RECESSIVE Exomiser Score: **0.998** Phenotype Score: **1.000** Variant Score: **0.999**

Variants contributing to score:
SPLICE_ACCEPTOR_VARIANT **chr1:g.94508455C>A [0/1]** rs767854160 (variation viewer)
Variant score: 0.999 CONTRIBUTING VARIANT
Transcripts:
ABCA4:ENST00000370225.3:c.3191-1G>T:p.?

Pathogenicity Data:
Best Score: 1.0
Mutation Taster: 1.000 (P)

Frequency Data:
Local: 0.0097%
gnomAD_E_NFE: 0.0018%

MISSENSE_VARIANT **chr1:g.94512532T>G [0/1]**
Variant score: 0.999 CONTRIBUTING VARIANT
Transcripts:
ABCA4:ENST00000370225.3:c.2861A>C:p.(Tyr954Ser)
ABCA4:ENST00000535735.1:c.2639A>C:p.(Tyr880Ser)

Pathogenicity Data:
Best Score: 1.0
Polyphen2: 0.765 (P)
Mutation Taster: 1.000 (P)
SIFT: 0.002 (D)

Frequency Data:
Local: 0.0097%

Figure S4. Screenshot of the Exomiser HTML output file (from the DEFAULT analysis) for patient P14 who was clinically diagnosed with cone-rod dystrophy and molecularly diagnosed with missense variant c.2861A>C:p.(Tyr954Ser) and splice acceptor variant c.3191-1G>T:p.? in *ABCA4* (Table S1).
